# Integration of Non-Coding RNA and mRNA Profiles Reveals the Mechanisms of Rumen Development Induced by Different Types of Diet in Calves

**DOI:** 10.3390/genes14051093

**Published:** 2023-05-16

**Authors:** Jie Wang, Huimei Fan, Mianying Li, Kaisen Zhao, Siqi Xia, Yang Chen, Jiahao Shao, Tao Tang, Xue Bai, Zheliang Liu, Yusheng Lu, Xiangrui Chen, Wenqiang Sun, Xianbo Jia, Songjia Lai

**Affiliations:** College of Animal Science and Technology, Sichuan Agricultural University, Chengdu 611130, China

**Keywords:** Chinese Holstein bull calves, different diet types, rumen tissue structure, gene expression

## Abstract

Selecting suitable feed types and understanding the gastrointestinal digestive mechanism are helpful for the growth and health of calves in intensive dairy farming. However, the effects on rumen development of changing the molecular genetic basis and the regulatory mechanism by using different feed types are still unclear. Nine 7-day-old Holstein bull calves were randomly divided into GF (concentrate), GFF (alfalfa: oat grass = 3:2) and TMR (concentrate: alfalfa grass: oat grass: water = 0.30:0.12:0.08:0.50) diet experiment groups. Rumen tissue and serum samples were collected for physiological and transcriptomic analysis after 80 days. The results showed that serum α-amylase content and ceruloplasmin activity were significantly higher in the TMR group, and Gene Ontology (GO) and Kyoto Encyclopedia of Genes and Genomes (KEGG) enrichment analysis ncRNAs and mRNAs were significantly enriched in the pathways of rumen epithelial development and stimulated rumen cell growth, including the Hippo signaling pathway, Wnt signaling pathway, thyroid hormone signaling pathway, ECM–receptor interaction and the absorption of protein and fat. The circRNAs/lncRNA-miRNAs-mRNA networks constructed, including novel_circ_0002471, novel_circ_0012104, TCONS_00946152, TCONS_00960915, bta-miR-11975, bta-miR-2890, PADI3 and CLEC6A, participated in metabolic pathways of lipid, immune system, oxidative stress and muscle development. In conclusion, the TMR diet could improve rumen digestive enzyme activities, stimulate rumen nutrient absorption and stimulate the DEGs related to energy homeostasis and microenvironment balance, and is thus better than the GF and GFF diets for promoting rumen growth and development.

## 1. Introduction

The dairy industry is an indispensable part of the national economy and is mainly based on natural grazing, semi-grazing and intensive farming. In the natural grazing and semi-grazing systems, due to the uncertainty of the ecosystem and environment, the DMI is low and ADF content is high in the diet, which reduces the efficiency of nutrient intake or absorption, and individual productivity cannot be fully developed [1]. Under forage-based livestock production systems, the quality and availability of feed determine the later growth and development of dairy cows [2]. Pasture is an important source of nutrition for calves. Selecting suitable feed types promotes the healthy growth of calves and reduces the feeding costs. Calves are fed solid feed after 7 days of life to increase productivity, fecundity and milk production, and to reduce the occurrence of disease [2,3]. The proportion and composition of solid feed can affect various aspects of development [4,5]. A high proportion of concentrated feed can improve ruminal starch digestibility, lower rumen pH and reduce the activity of the rumen papillae needed to absorb VFA [6,7,8]. The inclusion of dietary fiber through forage supplementation provides more physical stimulation, thereby enhancing the rumen volume, wall muscle content, motility and weight [9,10,11,12]. Therefore, with the rapid development of animal husbandry, determining whether different types of solid feed (including feed and concentrate) can provide certain nutrients for rumen growth and development is an urgent problem. Almost all studies to date on the effects of forage on rumen development have only been carried out at the level of nutrition, but the changes in the genetic basis and regulatory mechanism are still unclear and have never been studied.

Genomics takes all the nucleic acids of the organism as the research object. DNA, mRNA, lncRNA and other nucleic acid sequences are represented in the regulation of gene expression. The transcriptome is considered as the simplified research means for studying the genome, that is, the collection of all transcripts [13,14]. Therefore, RNA-seq is widely used to study the molecular mechanism of feed and to study the genes related to rumen growth and the development of ruminants, which play an important role in promoting rumen morphology and functional development [15,16]. miRNAs regulate the expression of target genes to maintain normal cellular homeostasis, digestion and nutrient absorption in the gastrointestinal tract [15,17]. lncRNA not only plays an important role in epigenetic inheritance, genomic imprinting and tissue cell formation, but also influences mRNA expression as an enhancer, promoter and transcriptional activator [18]. Studies have reported that being fed a high proportion of concentrate led to lncRNAs target genes activating PI3K-AKT, proteasome and HIF-1 signaling pathways, causing liver tissue inflammation and cell apoptosis, and aggravating SARA in dairy cows [15,19]. circRNA has a ring structure, is more stable than linear RNA and is rich in multiple binding sites of miRNA. It can regulate cell development, proliferation and differentiation by adsorbing miRNA and alleviating its inhibitory effect on mRNA in cells and tissues [20,21]. Zhang et al. showed that some candidate ceRNAs (cir_41, cir_115 and cir_171) might play key roles in immune and metabolic status in sheep [12].

Since the development of rumen in young animals is limited by their own physiological structure, the intake and type of solid feed are particularly important for nutrient absorption in rumen and the regulation of gene expression in epithelial cells to provide energy for themselves. In order to improve rumen growth and development, and the adult health and immunity of Holstein bulls before weaning, it is necessary to better understand the expression of ncRNAs and mRNAs in rumen tissues and the modifications involved in rumen growth- and development-related genes at the molecular level. At present, most studies on calves starter and abroad focus on nutrient levels and intestinal microorganisms, while there are few reports on the molecular genetic mechanism of rumen stimulation. Hence, the current study was designed to explore the serum immune factor content, digestive enzyme activities, rumen papilla size, ncRNAs and mRNAs regulatory mechanism, and their interactions under different feed types for the calf. The objective was to provide a comprehensive bioinformatics resource for selecting the most suitable feed type for rumen growth and development and the mechanism of ncRNA regulating rumen growth and development in calves.

## 2. Materials and Methods

### 2.1. Ethics Statement

This study was approved by and conducted in accordance with the ethical standards of the Institutional Animal Care and Use Committee of the College of Animal Science and Technology, Sichuan Agricultural University, Sichuan, 611130, China (20220236).

### 2.2. Animals and Feeding Strategy

The nine healthy Chinese Holstein bull calves (mean body weight, 41.58 ± 0.79 kg), 7 days old, were raised by the Mianyang Anzhou Hongfeng Dairy Cattle Breeding Co., Ltd. (Sichuan, China). They were randomly assigned to the GF, GFF and TMR groups, and each group was in a single pen. The GF, GFF and TMR groups were fed with “concentrate” (*n* = 3), “concentrate + hay” (*n* = 3), or a hay mixture (alfalfa: oat grass ratio = 3:2) and “concentrate: alfalfa grass: oat grass: water” = 0.30:0.12:0.08:0.50 (*n* = 3), respectively, with analysis after 80 days. The experimental diets were formulated according to the Chinese dairy cow feed standard (Table S1). Calves were given free access to both feed and water during the experimental time.

### 2.3. Detection of Rumen Papillae Height, Width and Number

All calves were slaughtered using electric-stunning slaughtering [22]. The back and abdomen tissue of rumen were collected. After fixation with 10% neutral formaldehyde, and after dehydration, pruning, embedding, sectioning, staining, sealing, etc., a Pannoramic 250 digital slice scanner produced by 3DHISTECH (Hungary) was used to collect images of the slices [23,24].

### 2.4. IgG Content and Digestive Enzyme Activity Detection

On the 80th day, before morning feeding, 5 mL of calf blood was collected by vacuuming the caudal vein with heparin sodium anticoagulant, and after standing for 30 min and centrifuging at 3000 r for 10 min to collect serum, it was stored at −80 °C in a refrigerator to determine serum indexes [15]. Serum samples were taken for IgG, α-amylase, ceruloplasmin, trypsin and lysozyme testing. An IgG ELISA kit was provided by Shanghai Zhuocai Biotechnology Co., Ltd. The AMS Kit, CP Assay Kit, Trypsin Assay Kit and LZM Kit were all provided by Nanjing Jiancheng Institute of Bioengineering.

### 2.5. Sample Collection and Transcriptomic Sequencing

#### 2.5.1. Total RNA Extraction from Calf Tissue

The collected rumen tissues were divided into 2 mL cryopreservation tubes and stored at −80 °C for further RNA extraction. The centrifuge was opened in advance and prechilled at 4 °C; after sterilization at high temperature, the mortar was wiped again with 75% alcohol, liquid nitrogen was prepared, the rumen tissue sample was ground into powder, an appropriate sample was put into a 1.5 mL EP tube, and 1 mL Trizol was added for total RNA extraction. The following steps were then carried out:

(1) After adding 200 μL chloroform, it was shaken up and down for 2 min until the chloroform fully reacted with the rumen tissue lysate; then, after standing on ice for 5 min, it was centrifuged at 4 °C for 15 min at 12,000 r;

(2) The supernatant of about 600 μL was sucked into a new 1.5 mL EP tube (the supernatant was sucked several times with a 200 μL pipette gun);

(3) After adding 600 μL isopropyl alcohol at a ratio of 1:1, it was shaken up and down until the reaction was complete; then, after standing on ice for 10 min, it was centrifuged at 12,000 r and 4 °C for 10 min;

(4) After quickly pouring out the supernatant and adding 1 mL of 75% ethanol prepared in advance, it was shaken upside down, and then centrifuged at 7500 r and 4 °C for 5 min;

(5) After pouring out the supernatant, a 200 μL pipette gun was used to suck out the remaining supernatant at the bottom;

(6) After instantaneous centrifugation, the remaining supernatant at the bottom was sucked out with a 100 μL pipette gun, and white precipitates could be seen;

(7) According to the amount of white precipitation, DEPC water was added to dissolve the RNA, and a 20 μL pipette gun was used to blow and mix. RNA integrity was determined by 1.5% agarose gel electrophoresis, and RNA quality was determined using a nucleic acid protein analyzer and an Agilent 2100 bioanalyzer. When the bands of the glue running results were between 28S and 18S without dragging, this indicated that the integrity of the proposed RNA was good. When the concentration of the measured RNA was 1.8 ≥ OD ≥ 2.0, this indicated that the purity was good, which met the requirements of the subsequent test. The concentration and purity of RNA (OD260/280) were detected using a Nanodrop instrument. Part of the extracted RNA was used for full transcriptomic sequencing and part was used for differential gene expression RT-qPCR verification.

#### 2.5.2. Transcriptomic Sequencing

The method of removing ribosomal RNA (circRNA library building to increase the process of removing linear RNA) was used to construct strain-specific libraries using Novogene [22]. Removing ribosomal RNA from total RNA, two strands were synthesized using RNA as a template. USER enzyme was used to degrade the second strand of cDNA containing U, and finally PCR amplification was performed to obtain the library [25]. First, Qubit was used for preliminary quantification, and the library was diluted to 1 ng/μL. Then, the Agilent 2100 BioAnalyzer was used to detect the insert size of the library [26]. The insert size was about 250–300 bp, in line with the expected Insert size. The effective concentration of the library was accurately quantified by qPCR. The effective concentration of the library was >2 nm and sequencing was performed using an Illumina PE150 [27].

### 2.6. Bioinformatics Analysis

#### 2.6.1. Quality Control

Trimmomatic Software was used to filter the Sequencing data to obtain clean readings [28]. The clean reads obtained by sequencing were compared to the reference genome using the comparison software, and the mapping rate was further analyzed [29].

#### 2.6.2. ncRNA Identity

Clean reads obtained by sequencing were compared with reference genomes using HISAT2 software to identify lncRNA [30]. The CuffCompare software was used for comparison with the known database, and the known transcripts in the database were filtered out. Finally, the coding potential of the screened new transcripts was predicted, and Novel_lncRNAs and mRNAs were obtained [31]. For the identity of miRNAs, clean reads of each sample were screened, and sRNA with lengths ranging from 18 to 35 nt were selected for subsequent analysis [32]. Bowtie was used to locate sRNA after length screening to the reference sequence and analyze the distribution of small RNA on the reference sequence [33]. The mapped reads were aligned on the reference sequence with the specified range sequence in miRBase to get the sRNA details for each sample match. Then, we integrated miRNAs prediction software miREvo and miR-deep2 to analyze new miRNAs [34,35,36]. For circRNA identity, we first analyzed the numbers in the CIGAR file. We scanned the numbers in the Sam file and paired chiastic clipping signals. Then, the Junction reads were filtered based on PEM and GT-AG signals, and finally, the DM algorithm was used to detect the Junction reads, which further filters and prevents the false positives caused by homologous gene similarity and repeated sequences [37].

#### 2.6.3. Quantification of Gene Expression Levels

StringTie was used to splice and quantify transcripts and genes using a network flow algorithm, and then the expression levels of all the known transcripts after alignment, splicing and screening, and the Novel_ncRNAs and mRNAs transcripts predicted, were quantified. Gene expression levels of mRNA and ncRNA were expressed as FPKM. FPKM (Fragments Per Kilobase of Transcript sequence Per Millions base pairs sequenced) is derived from a gene/transcript per kilobase length per million fragments count, which simultaneously corrects for the effect of sequencing depth and gene length on fragment counts, and is currently the most commonly used method for estimating gene expression levels [38].

#### 2.6.4. Differential Expression Analysis

Differential expression analysis of the three groups was performed using the DESeq R package (1.10.1) [39]. Genes with an adjusted *p*-value found by DESeq were assigned as differentially expressed. Significantly differential expression genes were screened based on the following criteria: corrected *p*-value < 0.05 and |(fold change)| > 2 [40].

#### 2.6.5. Function Annotations of RNA

GO enrichment analysis for host genes of differentially expressed ncRNAs was implemented by the GO-seq R package, in which gene length biases were corrected [41,42]. GO terms with corrected *p*-value < 0.05 were considered significantly enriched by differential gene expression. We used KOBAS software to test the statistical enrichment of differential expression genes or circRNA host genes in KEGG pathways [42,43]. The sequences of the DEGs were blast (blastx) to the genome of a related species (the protein interaction of which exists in the String data base: http://stringdb.org/ (accessed on 12 August 2022)) to get the predicted PPI of these DEGs. Then, the PPI of the DEGs was visualized in Cytoscape [15]. The interaction network was built and visually displayed using Cytoscape software based on the screening of cirRNA/lncRNA–miRNA–mRNA pairs [44].

### 2.7. Validation of RNA-Seq Results Analyzed by Quantitative PCR (qPCR)

To validate the repeatability of the RNA-seq analysis results, 18 candidate genes were randomly selected and evaluated by using qRT-PCR (for the primers, see Table S2). We performed qRT-PCR in a CFX96 Real-Time PCR Detection system (Bio-RadCo., Hercules, CA, USA) and detected RNA expression using SYBR Green Real-Time PCR Master Mix (Takara Co., Dalian, China). The relative expression levels were calculated using the 2^−∆∆Ct^ method and normalized to those of the reference genes ACTB and U6 [45,46].

### 2.8. The Data Statistical Analysis

IBM SPSS (27.0) software was used to analyze the experimental data by ANOVA. For quantitative variables, the data of the three groups were expressed as mean ± SD and compared using a *t*-test for statistical analysis. A value of *p* < 0.05 indicated a statistically significant difference [47].

## 3. Results

### 3.1. Development of Rumen Dorsal and Ventral Papillae

The results showed that the three different diets greatly affected the growth and development of rumen dorsal and ventral papillae in calves (Table 1). In particular, there were significant differences in the width and number of papillae in calves in the posterior abdominal sac between the three groups *(p <* 0.01). In the GF group, the height and width of the middle dorsal and middle ventral papillae of calves were significantly greater than those in the GFF and TMR groups (*(p <* 0.01).

### 3.2. Effects of IgG Content and Digestive Enzyme Activity

The serum content of IgG, α-amylase, ceruloplasmin, trypsin and lysozyme activity were measured (Table 2). The results showed that the α-amylase content and ceruloplasmin activity in the TMR group were significantly higher than those in the GF and GFF groups (*p* < 0.01). There were no significant differences in trypsin activity, lysozyme activity and IgG in the GF, GFF and TMR groups (*p* < 0.05).

### 3.3. Histopathological Examination (HE) for Pathological Change

**Figure 1:**
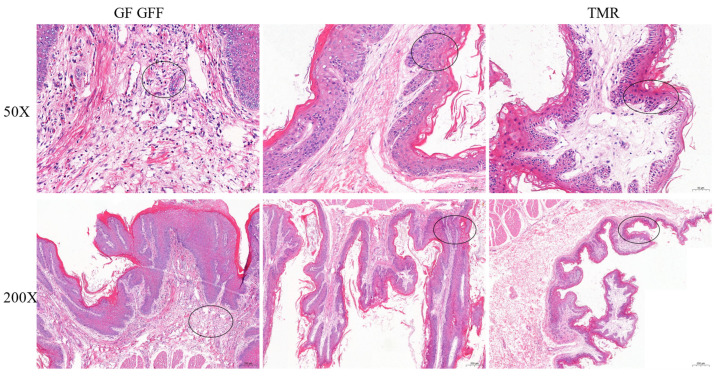
Dorsal tissue of rumen tissue samples stained with hematoxylin and eosin from Holstein cows fed different types of fodder. Note: GF: “concentrate”; GFF group: “concentrate + hay”; TMR: “TMR” type. Black circles indicate pathological features.

The tissue section method was used to determine if there was pathological change in the rumen of calves fed on the different types of fodder. In this study, HE revealed in the middle dorsum of rumen neutrophils and a few lymphocytes in the GF group. In the GFF group, it was more severe and the local epithelial necrosis penetrated into the lamina propria, the cells in the necrotic area disintegrated, and the nuclei were fragmented, accompanied by a large number of infiltrated neutrophils and a small amount of bleeding. In the TMR group, the keratinized layer of the mucosal layer was obvious, the morphology of cells in the middle layer and basal layer was intact, and the lamina propria was mildly edemic (Figure 1). The results showed that lesions were more severe in the GFF group, followed by the GF group, and less severe in the TMR group.

### 3.4. Overview of Identified ncRNA and mRNA

The Illumina HiSeq2500 sequencing platform was used to sequence 27 sample libraries, which generated a total of 72.18 GB of original data. After removing the reads with connectors and low quality in the original sequence, a total of 71.48 GB of clean reads were obtained, as shown in Table 3. The average base mismatch rate of the 27 sequenced libraries was 0.03%, the Q20 was higher than 97%, the Q30 was higher than 93%, and the GC content was 51.71%. The above sequencing quality control data showed that the experiment had high sequencing accuracy, a low base mismatch rate, reasonable distribution of base composition and good randomness in the sequencing process, which met the requirements of the later analysis. Then, we mapped the clean reads to the UMD 3.1 reference genome, and the alignment efficiency of ncRNA exceeded 90.63%.

**Figure 2:**
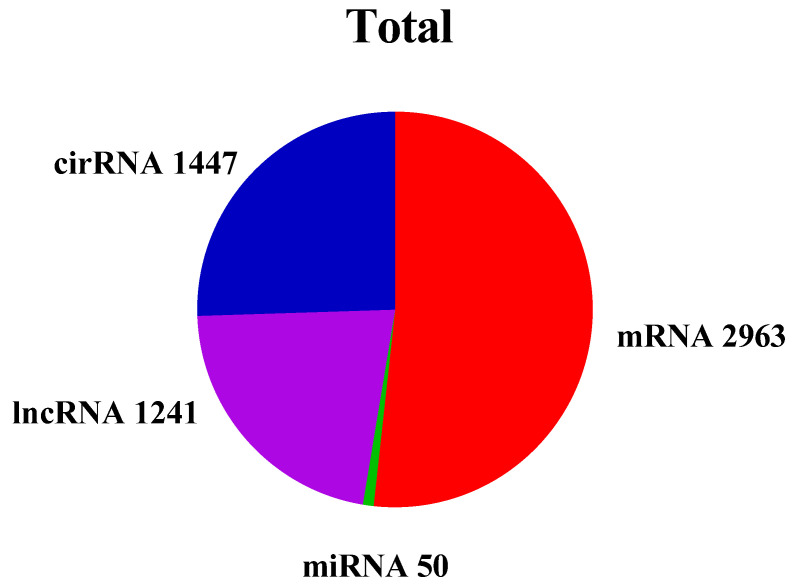
Total numbers of RNA in the rumen tissue.

**Figure 3:**
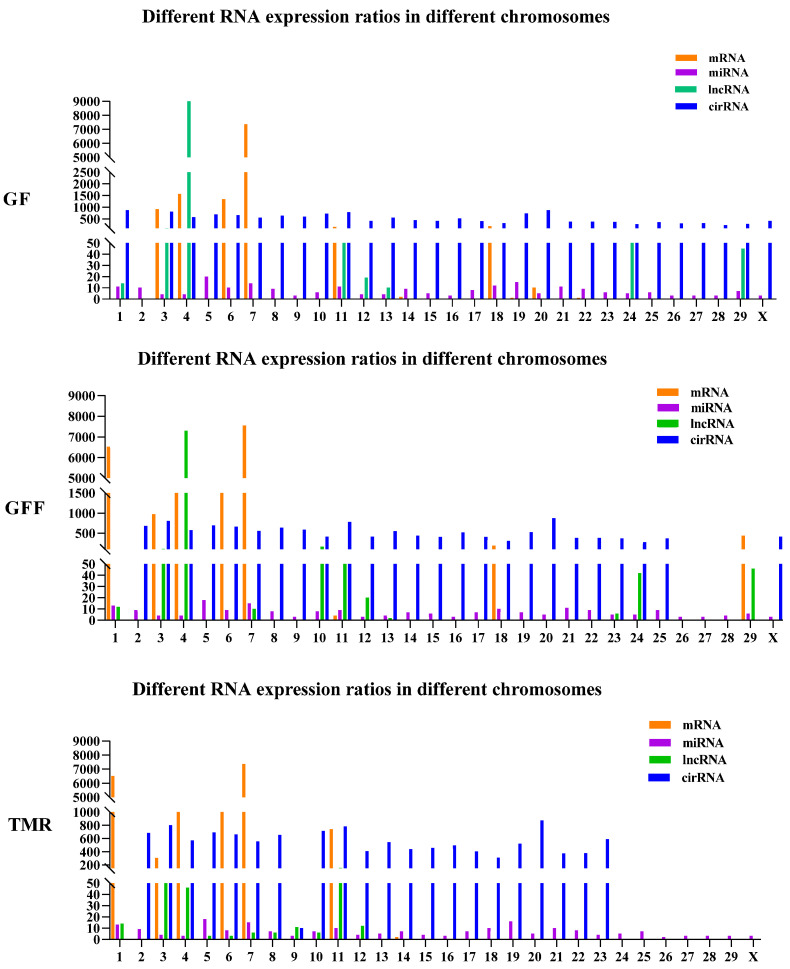
ncRNAs position of chromosome of rumen tissue. Different RNA expression ratios in different chromosomes; different colors represent different RNA types.

To identify ncRNA in calf rumen, transcripts were filtered by following a rigorous set of criteria. Finally, 50 miRNA, 1241 lncRNA and 1447 circRNA were obtained (Figure 2). To assess the distribution of the putative RNA, we analyzed the RNA expression ratio across the chromosomes. The putative RNAs were not equally distributed across the rumen chromosomes in Figure 3. Most mRNA were found on chromosome 1 and 7, miRNA were found on chromosome 5, whereas lncRNA were found on chromosome 7. In addition, most circRNA were found on chromosome 20 and X.

### 3.5. Expression Patterns of mRNAs and ncRNAs

**Figure 4:**
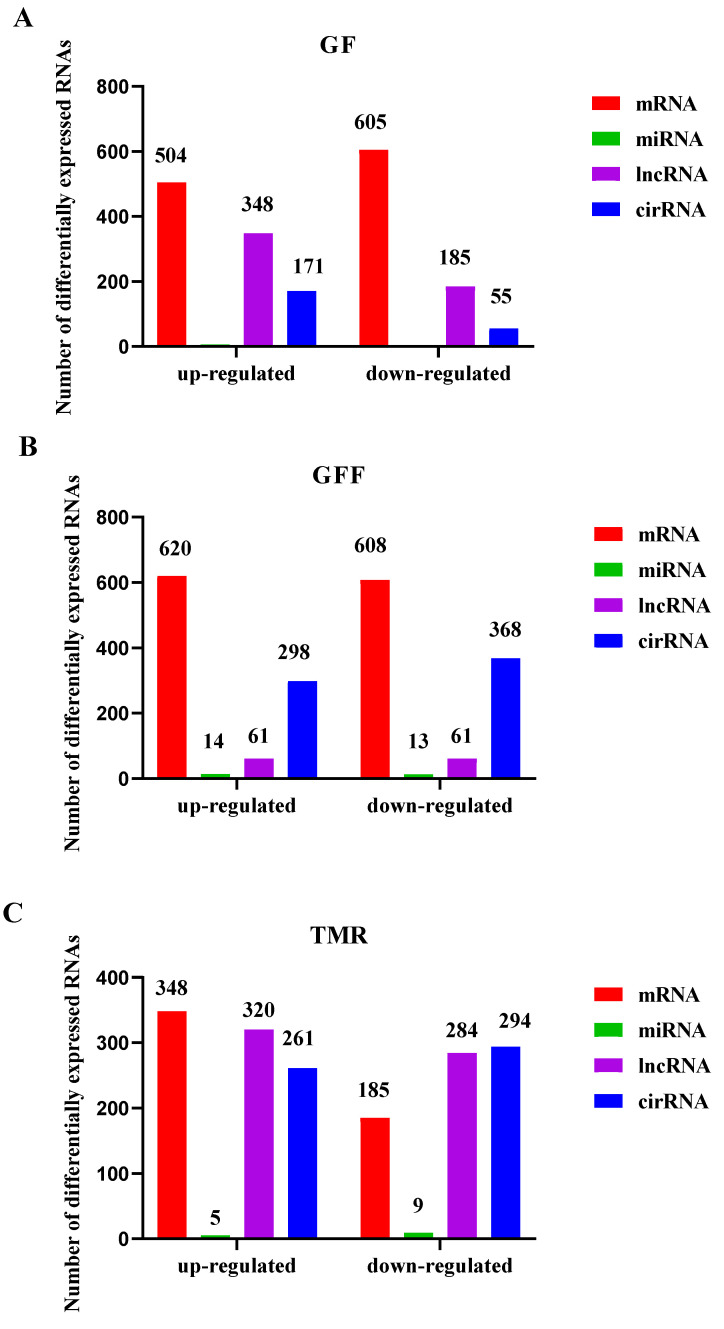
The number of differentially expressed RNAs represented by histograms. Schematic representation of the expressed RNA in GF (**A**), GFF (**B**) and TMR (**C**) groups.

To determine whether ncRNAs are involved in rumen growth and development, the DE ncRNAs and mRNAs from the different feed type groups were visualized using a histogram (Figure 4). There were 1447 DE-circRNAs (730 up-regulated and 717 down-regulated), 1259 differentially expressed DE-lncRNAs (729 up-regulated and 530 down-regulated), 49 DE-miRNAs (23 up-regulated and 25 down-regulated) and 2870 DEGs (1472 up-regulated and 1398 down-regulated) that were identified based on the criteria of *p*-value < 0.05 and |(fold change)| ≥ 1 by comparisons of samples collected from rumen (Figure 4A–C).

### 3.6. Differentially Expressed ncRNAs and mRNA

**Figure 5:**
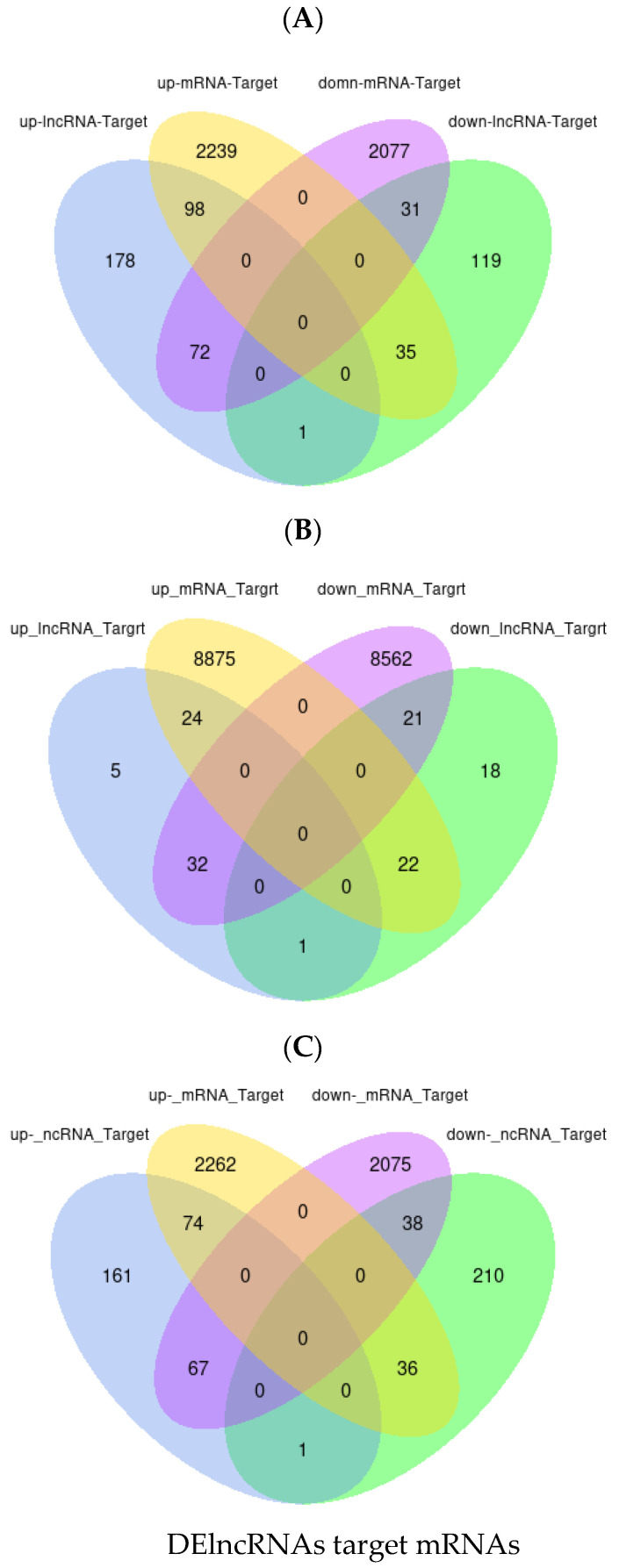
The co-expression intersections of lncRNAs target genes shown in Venn diagrams. The number of common DEGs and lncRNAs in GF (**A**), GFF (**B**) and TMR groups (**C**). DEG: differentially expressed genes; lncRNAs: differentially expressed long non-coding RNA.

The study identified the overlap between lncRNA target genes and DE mRNAs (Figure 5A–C). It was found that the relationships between XLOC_588071 and SLC51A, ARNTL2, CFAP299, XLOC_1002614 and C9, HNF4A, XLOC_609407, XLOC_994154 and ANXA13, XLOC_589694 and MYH11, LDB3 were co-expressed, which indicated that those lncRNAs are potential regulators.

**Figure 6:**
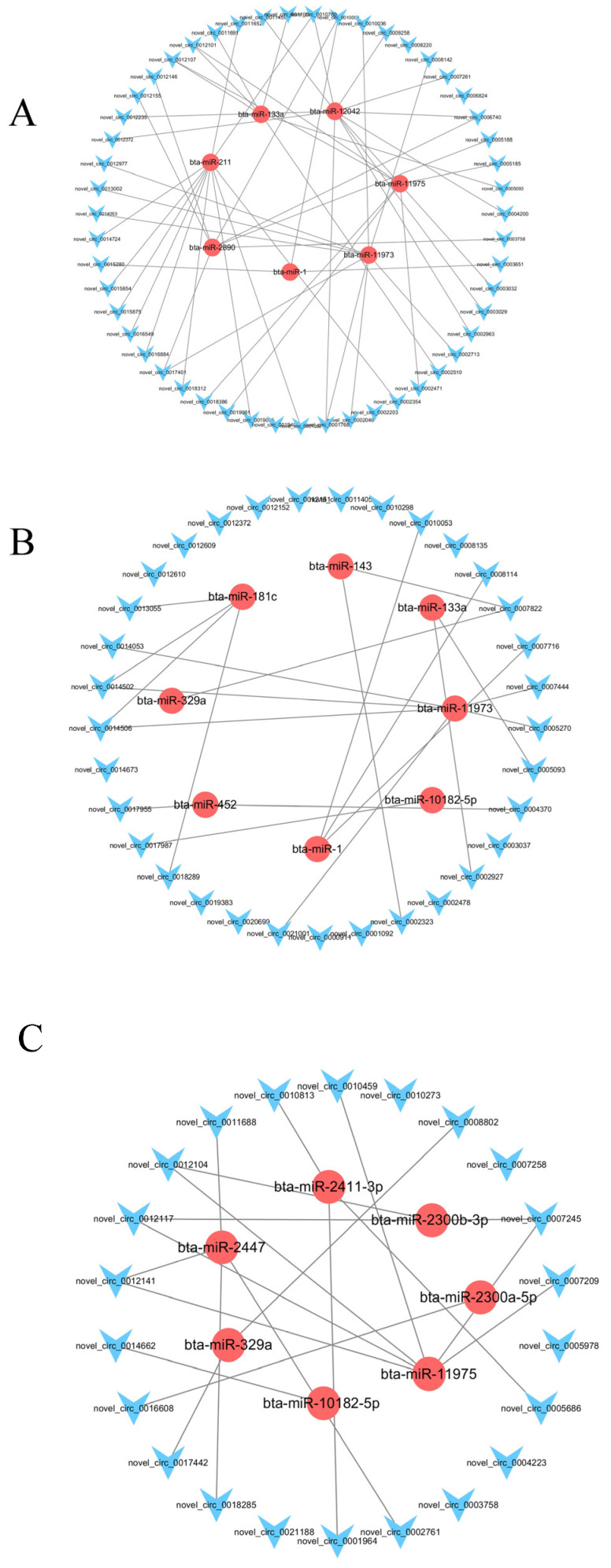
Diagrams of co-expressed networks were constructed for circRNAs-miRNAs in GF (**A**), GFF (**B**) and TMR (**C**). Light blue and diamond nodes represent circRNAs; light red and circular nodes represent miRNAs.

Co-expression network analysis was performed on the selected differentially expressed circRNAs related to rumen growth and development and their targeted miRNAs. In the interaction network, there were four specific differentially expressed cirRNAs and their interacting miRNAs. According to the degree value, novel_circ_0019095, novel_circ_0011652, novel_circ_0016549 and bta-miR-211, novel_440, novel_circ_0012141, novel_circ_0012117 and bta-miR-2300b-3p, novel_circ_0008114 and bta-miR-1, etc., played an important regulatory role in the network (Figure 6 and Table 4).

### 3.7. Differentially Expressed ncRNAs and mRNA Functional Enrichment Analysis

**Figure 7:**
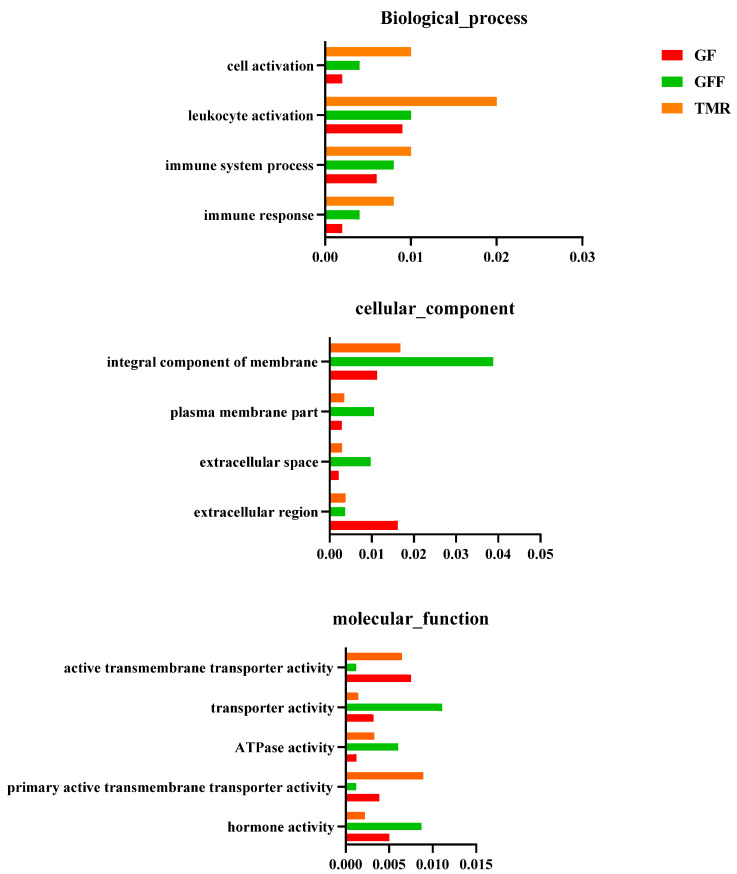
Function enrichment analysis for the source genes of ncRNAs. GO distribution of source genes. The top 10 enriched KEGG pathways ranked by (*p <* 0.05) are shown.

We further explored the potential regulatory roles of ncRNAs in rumen growth, which predicted the functions of ncRNAs using GO. The study shows that enrichment analysis based on the co-expression of lncRNAs (Figure 7 and Table S3). The most significantly enriched in the three groups were immune response, immune system process, lymphocyte activation, extracellular region and extracellular space, hormone activity and Ras GTPase binding biological processes.

**Figure 8:**
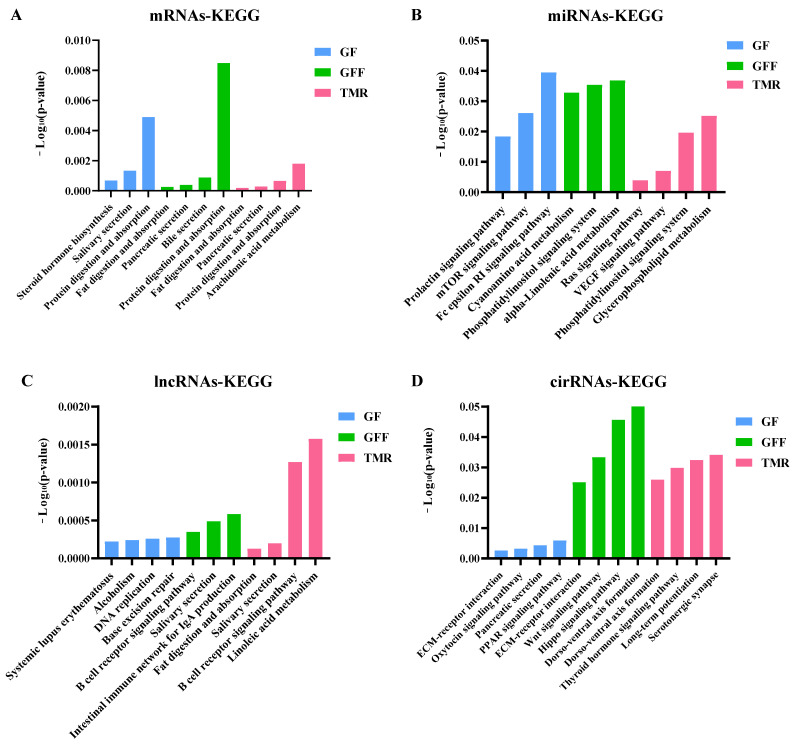
Function enrichment analysis for the source genes of ncRNAs. KEGG classification of source genes ((**A**): mRNAs, (**B**): miRNAs, (**C**): lncRNAs and (**D**): circRNAs). The top 10 enriched KEGG pathways ranked by (*p <* 0.05) were shown.

The study revealed the most enriched pathways for mRNAs and circRNA/lncRNA-miRNA-mRNAs using *p <* 0.05. Interestingly, the ncRNA target genes exhibited KEGG enrichment patterns similar to those of the DE mRNAs (Figure 8 and Table S4). The most enriched pathways were nutrient digestion and absorption (pathways in fat digestion and absorption, protein digestion and absorption, glycerophospholipid metabolism, α-linolenic acid metabolism, ABC transporter pathways and dorso–ventral axis formation) and signaling molecule and interaction pathways (NF-kappa B signaling pathway, intestinal immune network for IgA production, Wnt signaling pathway, Hippo signaling pathway, extracellular matrix (ECM)–receptor interaction and thyroid hormone signaling pathway) in the GF and TMR groups. In the GFF group, the most significant pathways were pathways in steroid hormone biosynthesis, cytokine–cytokine receptor interactions, systemic lupus erythematosus, alcoholism, DNA replication, base excision repair and arrhythmogenic right ventricular cardiomyopathy (ARVC). These genes are related to energy production and disease.

### 3.8. Construction of a Potential lncRNA/circRNA-miRNA-mRNA Regulatory Network

**Figure 9:**
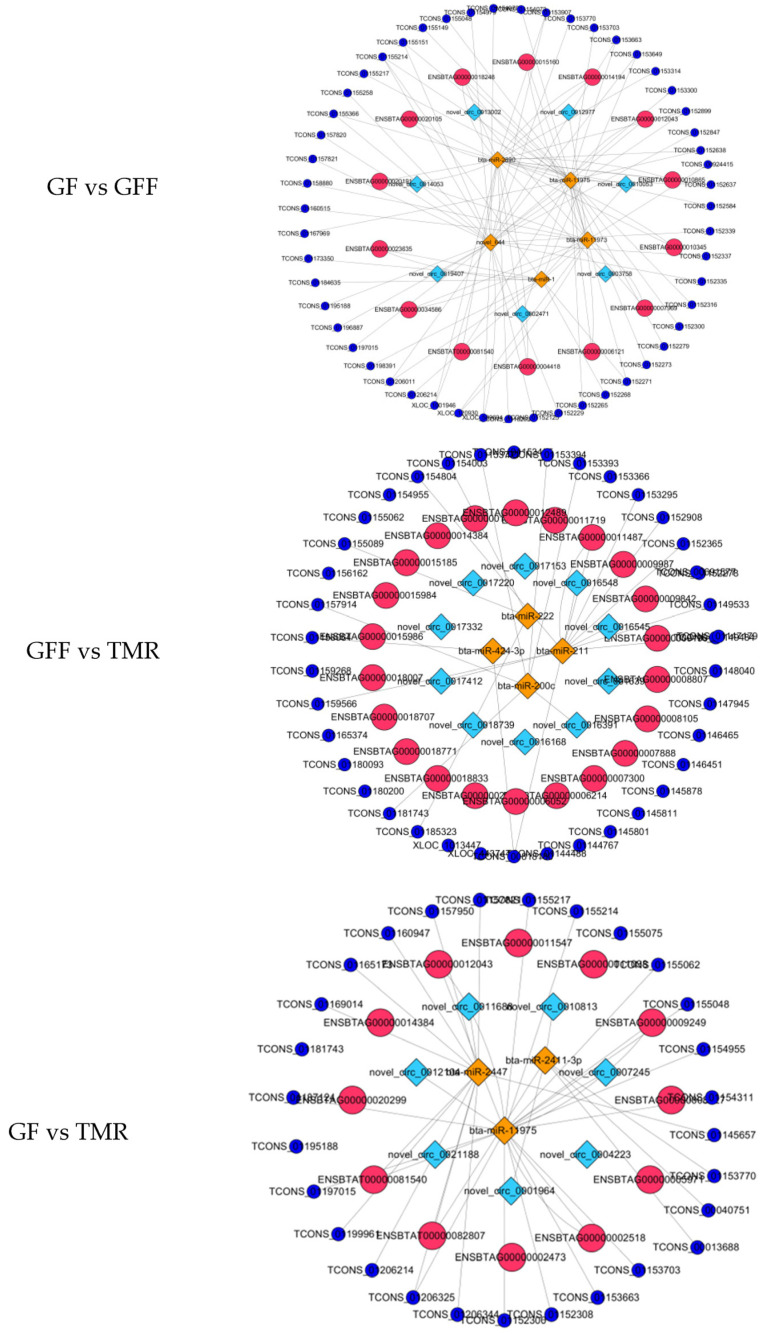
Co-expressed network construction. Orange and diamond nodes represent miRNAs; light blue and diamond nodes represent circRNAs; light red and circular nodes represent mRNAs; indigo and circular nodes represent lncRNAs.

The results indicate that differentially expressed growth- and development-related genes in each group may participate in multiple different ceRNA networks [48]. More importantly, this study found that miRNAs may participate in different ceRNA networks in different rumen development nodes. For example, in the groups of GF vs. GFF, bta-miR-11973 involves a total of 3 mRNAs, 33 lncRNAs and 3 circRNAs in the network. However, in the GF vs. TMR groups, bta-miR-11973 may also be regulated by 14 lncRNAs and 2 circRNAs (Figure 9 and Table S5). The results present the regulatory relationships between ncRNAs and mRNAs in the processes of rumen growth and development.

### 3.9. Validation of DE Genes by qRT-PCR

**Figure 10:**
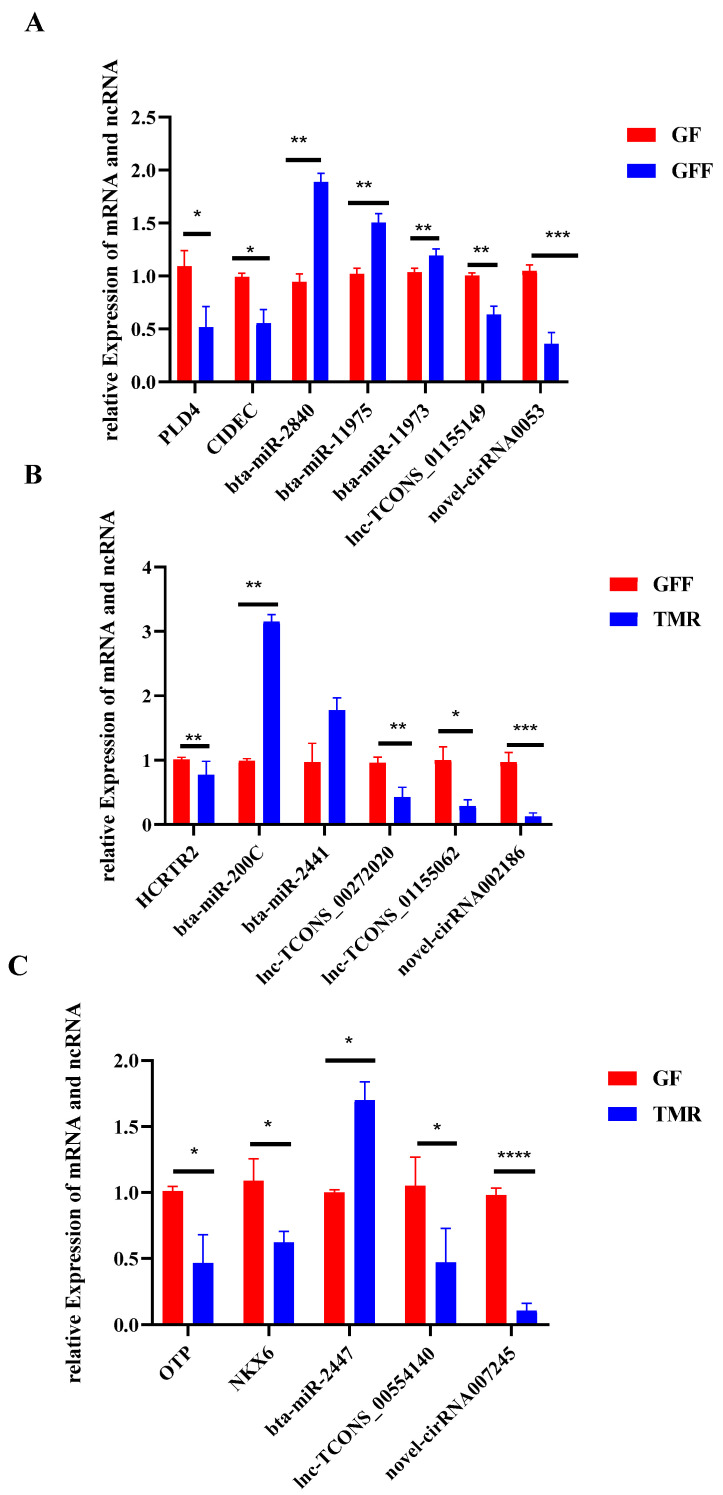
Expression levels of genes validated by RT-qPCR. The GADPH and U6 were used as internal controls, and the relative quantity of gene expression of each gene was calculated with the comparative 2^−∆∆Ct^ method. Values (RT-qPCR) shown are mean with SD. * *p* < 0.05; ** *p* < 0.01, *** *p* < 0.001, and **** *p* < 0.0001. (**A**): GF vs. GFF group; (**B**): GFF vs. TMR group; (**C**): GF vs. TMR group.

To ensure the accuracy and validity of the sequencing results, 18 genes (ncRNAs and mRNAs) were randomly selected and verified by qRT-PCR. Compared with the RNA-seq data, similar expression trends for qRT-PCR were observed, showing the strong consistency between the qRT-PCR and RNA-seq data (Figure 10).

## 4. Discussion

The growth of the rumen papillae was the main manifestation of the physiological development of the rumen [49]. Most obviously, the height and width of the ruminal papillae of calves in the GF group were significantly greater than those in other groups. High concentrate diets contain a large amount of starch that could be converted into butyrate VFA by microorganisms to stimulate the growth of the rumen papillae, increasing the area of the papillae [50,51]. In the current study, the effective surface area of the rumen papilla fed high-concentrate diet was enlarged, which promoted nutrient absorption by the calves. Digestive enzyme activity plays an important role in the digestion of rumen nutrients, the regulation of pancreatic secretion and the promotion of pancreatic enzyme synthesis, and it is a good indicator for judging feed efficiency and the growth and development of calves [52]. In this experiment, we found that the TMR group diet increased the rumen α-amylase content and ceruloplasmin activity of calves, which were beneficial to the intake, digestion and utilization of solid feed, thus laying a good foundation for the intake of solid feed for calves [53]. Appropriate intake of concentrate could stimulate ruminal papillae growth and development. Likewise, a certain amount of roughage was also important for maintaining the degree of keratinization of the rumen epithelium [52]. Current results indicated that the TMR-type diet was more conducive to growth and development in calves.

The quality control and alignment efficiency results indicated that a successful library was constructed with high sequencing quality. In the study, 2935 mRNA, 50 miRNA, 1241 lncRNA and 3508 circRNA were obtained from the clean data. The results indicated that ncRNA play an important role in rumen growth and development at the transcriptional level. Furthermore, RT-qPCR was performed to confirm the reliability of sequencing data [54]. In order to understand the potential functions of the ncRNAs identified, functional enrichment analyses on the target genes of DE ncRNAs were performed. It was obvious that the functional GO enrichment of these DEGs was mainly related to the immune response. Fueling immune response is an energetically expensive process [55]. The transfer of nutrients from growth- to immune-related processes may increase maintenance requirements in animals during the immune response [12,56]. Therefore, the results of GO enrichment of these DEGs led to speculation that feeding calves with different feed types might result in less energy being consumed for combating systemic inflammation, and hence more efficient utilization of nutrients for growth and protein accretion.

The results of KEGG pathway analysis showed that the Hippo signaling pathway, Wnt signaling pathway, thyroid hormone signaling pathway and ECM–receptor interaction were significantly enriched in the TMR group. Those signaling molecule pathways are vital to cell differentiation, cell proliferation, regulated development and homeostasis, and rumen epithelial development [57,58,59,60]. In the present study, the TMR group might induce the differentiation of rumen cells and promote the maturation of rumen cells by signaling molecules and interaction. Systemic lupus erythematosus, DNA replication and alcoholism metabolic pathways were highly expressed in the GFF group in particular. Subsequently, we screened DEGs related to these pathways, such as HIST1H2BJ, HIST1H2AG, HIST1H2BB, HIST1H2BD and H4. Importantly, H4 binds to DNA to act in DNA replication and DNA repair by activating B-cell receptor signaling and autoantibodies. At the same time, the abnormal expression of DEGs was involved in the occurrence of cervical cancer [61]. It is worth mentioning that the Ras signaling pathway and Fc γ R-mediated phagocytosis single pathway were highly expressed in the GF and GFF groups. The literature reports that supplementation with high-concentrate diet, which activates the immune system, requires considerable energy, and the immune response might be an important factor contributing to the poorer feed efficiency and decreased performance of finished cattle [42,55,62,63]. Consistent with the results of this study, the literature indicates that feeding high-concentration concentrate to calves could trigger a series of inflammatory reactions, disorder of the immune system and glucose and lipid metabolism, which is not conducive to rumen growth and development. The above results indicated that the TMR group diet is more beneficial to rumen growth and development in calves.

Finally, to further understand how ncRNAs regulate rumen development, we constructed circRNAs/lncRNAs-miRNAs-mRNAs interaction networks based on competing ceRNAs to reveal the main functions of these ncRNAs. In the interaction network of the TMR group, some circRNAs containing several target sites of miRNAs (novel_circ_0002471, novel_circ_0012104 and bta-miR-11975) were found. Simultaneously, nine targeted key genes (PADI3, CLEC6A, FEV, MGLL, CACNG2, PLA2G2E, PLEKHS1, PSD and FHL3) were identified from five miRNAs (bta-miR-11975, bta-miR-1, bta-miR-2890, bta-miR-11974 bta-miR-211 and bta-miR-486), which participate in lipid, immune system, oxidative stress and muscle development and in the immune system. In addition, the highly expressed TCONS_00946152, TCONS_00960915 could act as ceRNAs to adsorb bta-miR-11975, and mainly participate in energy homeostasis and microenvironment balance by participating in the phosphatidylinositol signaling system, protein digestion and absorption, and arachidonic acid metabolism. The results might be related to the development of rumen, but the specific mechanism of action needs to be further investigated.

The TMR-type diet for calves in this experiment promoted enhanced rumen growth and development (as determined by α-amylase content and ceruloplasmin activity). In the rumen tissue from calves fed with the different type diets, the target genes of differentially expressed ncRNAs were significantly enriched in pathways closely related to rumen epithelial development and stimulated rumen cell growth, including the Hippo signaling pathway, Wnt signaling pathway, thyroid hormone signaling pathway and ECM–receptor interaction. Some target genes, including novel_circ_0002471, novel_circ_0012104, TCONS_00946152, TCONS_00960915, bta-miR-11975, bta-miR-2890, PADI3 and CLEC6A, play important roles in rumen development in calves. These target genes and pathways might be involved in the absorption and transport of protein and fat, further improving the growth performance of calves. The pathways and affected genes might serve as markers for further investigation into the mechanisms regulating growth and impacting rumen development, and provide favorable conditions for calf growth.

## 5. Conclusions

In this study, the GF group diet significantly increased the area of rumen papillae, the miRNAs and lncRNAs target genes were highly expressed in inflammatory-response-related pathways, and single concentrate could cause a series of inflammatory reactions, which were not conducive to rumen growth and development. lncRNAs target genes in the GFF group were abnormally expressed during energy production and consumption, which reduced the feed utilization rate of calves, and led to immune system and lipid metabolism disorders in calves. The TMR diet was beneficial to maintain the keratinization degree of rumen epithelium, provide abundant conjugated linoleic acid, and induce rumen cell differentiation and promote rumen cell maturation through signaling molecule interaction. Therefore, in order to improve the rumen growth and development of calves and the long-term stable development of animal husbandry, the TMR feeding strategy is recommended. There are some limitations to this study. Considering animal welfare and the 3Rs principle, the number of calf samples used in this study was limited, which made the biological repetition of this experiment lower, but it still provides a comprehensive information resource for future research on related aspects. Therefore, in vitro experiments are needed to further improve relevant studies.

## Figures and Tables

**Table 1:** The development of papillae of ruminal dorsal and abdominal sacs (mean ± SD).

Terms	GF			GFF			TMR		
	Papillae Height (μm)	Papillae Width (μm)	Papillae Number	Papillae Height (μm)	Papillae Width (μm)	Papillae Number	Papillae Height (μm)	Papillae Width (μm)	Nipple Number
anterior dorsum	1906.43 ± 1257.35 ^a^	400.66 ± 149.18 ^a^	6.89 ± 1.24	1501.97 ± 1060.40 ^b^	274.47 ± 96.73 ^b^	8.61 ± 3.95	1540.82 ± 933.12 ^b^	269.94 ± 102.15 ^b^	6.83 ± 2.22
middle dorsum	2128.66 ± 1451.40 ^a^	419.27 ± 174.35 ^a^	4.50 ± 1.62 ^a^	1922.92 ± 1423.43 ^a^	333.18 ± 120.577 ^b^	7.72 ± 3.68 ^a^	1171.69 ± 587.37 ^b^	317.49 ± 135.73 ^b^	9.28 ± 1.18 ^b^
back dorsum	1483.10 ± 1174.22 ^a^	316.11 ± 125.19	6.39 ± 1.80	2217.58 ± 1398.27 ^b^	341.29 ± 255.09	7.11 ± 2.87	1725.35 ± 870.44 ^b^	319.22 ± 87.99	8.50 ± 1.30
anterior abdomen	1326.79 ± 1116.87 ^a^	330.39 ± 153.94	5.11 ± 1.92 ^a^	2286.27 ± 1329.40 ^a^	306.41 ± 153.94	7.67 ± 1.44 ^a^	1907.41 ± 1485.78 ^b^	262.73 ± 92.01	7.17 ± 1.89 ^b^
middle abdomen	1668.26 ± 1162.20 ^a^	315.91 ± 125.38 ^a^	4.50 ± 1.12 ^a^	2198.94 ± 1309.87 ^b^	44.66 ± 87.85 ^b^	6.56 ± 2.60 ^ab^	1631.82 ± 899.75 ^b^	244.66 ± 87.85 ^b^	8.38 ± 2.43 ^b^
posterior abdomen	2037.77 ± 1566.81 ^a^	336.90 ± 146.27 ^a^	4.28 ± 0.83 ^a^	1726.60 ± 1211.84 ^a^	275.17 ± 88.79 ^b^	6.56 ± 2.13 ^b^	1025.13 ± 649.82 ^b^	224.38 ± 71.08	9.45 ± 2.51

Note: Values with superscript letters ^a^, ^b^ and ^ab^ are significantly different across lines (*p* < 0.05). First, all the averages were arranged in descending order, and then the largest marked with the letter ^a^. This average was compared with the other two sets of averages and marked with the letter ^a^ if the difference was not significant, and significant differences were marked with the letter ^b^. The mean labeled ^b^ was taken as the standard and compared to the mean of the other group, without any letters if the difference was not significant, or with ab if it was in the same line as ^b^.

**Table 2:** Effects of digestive enzymes and IgG on rumen growth and development in calves (mean ± SD).

Fluency Index	GF	GFF	TMR
	Mean ± SD (%)	Mean ± SD (%)	Mean ± SD (%)
α-amylase content (nmol/mL)	35.65 ± 3.40 ^b^	57.96 ± 9.68 ^b^	38.55 ± 10.68 ^a^
Ceruloplasmin activity (U/L)	9.19 ± 0.06 ^b^	8.81 ± 1.65 ^b^	12.73 ± 0.13 ^a^
Trypsin activity (U/mL)	213.56 ± 187.52	89.56 ± 50.97	265.22 ± 173.63
Lysozyme content (μg/mL)	1.50 ± 0.09	1.72 ± 0.17	1.62 ± 0.43
IgG (mg/mL)	0.56 ± 0.11	0.58 ± 0.08	0.60 ± 0.32

Note: Values with superscript letters ^a^ and ^b^ are significantly different across lines (*p* < 0.05). First, all the averages were arranged in descending order, and then the largest marked with the letter ^a^. This average was compared with the other two sets of averages and marked with the letter ^a^ if the difference was not significant, and significant differences were marked with the letter ^b^.

**Table 3:** Quality control of mRNA and long-chain non-coding RNA sequencing.

Sample Name	Raw Reads	Clean Reads	Clean Bases	Error Rate (%)	Q20 (%)	Q30 (%)	GC Content (%)
GF_R_1	95151460	94466002	12.79G	0.03	97.58	93.48	52.04
GF_R_2	83295944	82575946	14.17G	0.03	97.62	93.66	55.92
GF_R_3	99995438	99117250	12.39G	0.03	97.62	93.60	52.20
GFF_R_1	81517354	80087068	12.01G	0.03	97.48	93.33	51.38
GFF_R_2	79648920	78647860	11.80G	0.03	97.59	93.55	52.30
GFF_R_3	91568644	90891982	13.63G	0.03	97.72	93.81	49.97
TMR_R_1	85349140	84721348	12.71G	0.03	97.60	93.48	48.89
TMR_R_2	85825104	85003696	12.75G	0.03	97.70	93.81	52.33
TMR_R_3	86443542	85679884	12.85G	0.03	97.71	93.78	50.36

**Table 4:** ncRNAs and their potential target genes involved in rumen development.

Group	miRNAs	circRNAs	lncRNAs	mRNAs
GF vs. GFF	bta-miR-11973	novel_circ_0014053, novel_circ_0012977, novel_circ_0013002	XLOC_432876, XLOC_480762, XLOC_1012137, XLOC_098896	MAL, PLD4, CIDEC,
	bta-miR-1	novel_circ_0010053	XLOC_511980, XLOC_1005675, XLOC_1005790, XLOC_062658, XLOC_122211	CLEC6A
	bta-miR-2890	novel_circ_0003758, novel_circ_0010053	XLOC_1001781, XLOC_999381, XLOC_654245, XLOC_998773	FEV, MGLL, CACNG2, PLA2G2E
GFF vs. TMR	bta-miR-200c	novel_circ_0021078, novel_circ_0021086, novel_circ_0016391, novel_circ_0008818,	XLOC_514666, XLOC_223927, XLOC_546291, XLOC_005658	XLOC_398026, XLOC_1006227, XLOC_629685, XLOC_1001314XLOC_1013447
	bta-miR-211	novel_circ_0015818, novel_circ_0015854, novel_circ_0011652, novel_circ_0016548, novel_circ_0011061	GTPBP4, XLOC_1006044, XLOC_998134.SPDYA	XLOC_253297, XLOC_443744, PLEKHS1, XLOC_830139
GF vs. TMR	bta-miR-11975	novel_circ_0012104, novel_circ_0007245	CDCP1, EFHB, XLOC_996619, XLOC_996619, XLOC_1003325, LYRM1, XLOC_1001987, FBXL19	NKX6-1, OTP, PADI3
	bta-miR-2411-3p	novel_circ_0010813, novel_circ_0001964	XLOC_999378, SLX4IP, SLX4IP, XLOC_59297	XLOC_1005215, XLOC_830139

## Data Availability

The original data are stored in GSA, number: PRJCA016969.

## Supplementary Materials

The following supporting information can be downloaded at: https://www.mdpi.com/article/10.3390/genes14051093/s1, Table S1: Feed formula; Table S2: Genes’ primer information; Table S3: GO analysis of differentially expressed ncRNAs; Table S4: KEGG analysis of differentially expressed ncRNAs; Table S5: Expression of lncRNAs/circRNAs-miRNAs-mRNAs.

## List of Abbreviations

**Item**	**Unit**
GF	Concentrate
GFF	Concentrate + hay
TMR	Concentrate + alfalfa grass + oat grass: water
mRNA	Messenger ribonucleic acid
miRNA	MicroRNA
lncRNA	Long non-coding RNA
circRNA	Circular RNA
ncRNA	Non-coding RNA
mRNAs	Differentially expressed mRNA
miRNAs	Differentially expressed miRNA
lncRNAs	Differentially expressed lncRNA
circRNAs	Differentially expressed circRNA
ncRNAs	Differentially expressed non-coding RNA
SNP	Single Nucleotide Polymorphisms
InDel	Insertion–deletion
GO	Gene Ontology
KEGG	Kyoto Encyclopedia of Genes and Genomes
CeRNAs	Competing endogenous RNAs
PPI	Protein–protein interaction
qRT-PCR	Quantitative real-time PCR
SD	Standard deviation
DMI	Dry matter intake
ADF	Acid detergent fiber
VFA	Volatile fatty acid
PH	Potential of hydrogen
RNA-seq	RNA sequencing
IgG	Immunoglobulin G
AMS	α-amylase
CP	Ceruloplasmin
LZM	Lysozyme
SARA	Subacute ruminal acidosis
MRE	miRNA response element
mean ± SD	mean ± standard deviation
